# Longitudinal Peripheral Immunophenotyping and Rituximab Response in Primary Membranous Nephropathy: A Prospective Cohort Study

**DOI:** 10.3390/ijms27219956

**Published:** 2026-11-06

**Authors:** Christos Georgopoulos, Lefkothea Dova, Eleni Stamellou, Anila Duni, Georgios Vartholomatos, George Liapis, Vasileios Patsiogiannis, Ekaterini Siomou, Haralampos Milionis, Evangelia Dounousi

**Affiliations:** 1 Department of Nephrology, University of Ioannina and University Hospital of Ioannina, Stavrou Niarchou Avenue, 45500 Ioannina, Greece; georgopoulosch@gmail.com (C.G.); stamellou.eleni@googlemail.com (E.S.); anikristduni@yahoo.com (A.D.); 2 Laboratory of Hematology—Unit of Molecular Biology, University Hospital of Ioannina, 45500 Ioannina, Greece; thea_dova@yahoo.gr (L.D.); gvarthol@gmail.com (G.V.); 3 First Pathology Laboratory, General Hospital of Athens “Laiko″, 11527 Athens, Greece; gliapis@gmail.com; 4 Department of Radiology, University Hospital of Ioannina, 45500 Ioannina, Greece; vpatsiogiannis@gmail.com; 5 Department of Pediatrics, University Hospital of Ioannina, 45500 Ioannina, Greece; eksiomou@yahoo.gr; 6 First Department of Internal Medicine, University Hospital of Ioannina, 45500 Ioannina, Greece; hmilioni@uoi.gr

**Keywords:** B-lymphocytes, biomarkers, flow cytometry, glomerulonephritis, membranous, rituximab

## Abstract

Rituximab (RTX) is a first-line option for primary membranous nephropathy (pMN), but many patients fail to respond for reasons that conventional markers only partially explain. We assessed whether longitudinal peripheral immunophenotyping could identify cellular biomarkers of response. In a prospective single-center cohort of 35 patients with pMN treated with standard two-dose RTX, flow cytometry of B-cell, T-cell and monocyte compartments, the systemic inflammation response index (SIRI) and phospholipase A2 receptor antibody were assessed at baseline and months 3, 6 and 12. Within a mean of 7.48 ± 2.4 months, 23 patients (65.7%) achieved either partial or complete remission. By month three, responders showed deeper B-cell depletion, higher CD4+CD25+FoxP3+ T cells, fewer intermediate monocytes and a lower SIRI. On multivariate analysis, month-3 depletion of total CD3−CD19+ B cells below 0.5% (odds ratio 5.8, 95% confidence interval 1.1–31.1) was associated with remission, and each 0.1-unit increase in month-3 SIRI (odds ratio 0.44, 95% confidence interval 0.22–0.91) with lower odds of remission. In a hypothesis-generating analysis, a model combining both had an apparent area under the curve of 0.87. Longitudinal immunophenotyping identifies candidate biomarkers of RTX response in pMN that require multicenter validation.

## 1. Introductions

Primary membranous nephropathy (pMN) is the leading cause of nephrotic syndrome in non-diabetic adults and is an antibody-mediated autoimmune glomerular disease. In most patients, circulating autoantibodies target the podocyte M-type phospholipase A2 receptor (PLA2R1) [1], whereas a minority harbor antibodies against thrombospondin type-1 domain-containing 7A [2] or one of a growing list of more recently described podocyte antigens [3]. These autoantibodies deposit in the subepithelial space, activate complement and injure the glomerular filtration barrier, causing proteinuria, whose course ranges from spontaneous remission to progressive, kidney-function-threatening disease [1].

The 2021 Kidney Disease: Improving Global Outcomes (KDIGO) guideline reframed the management of primary MN around the risk of progressive loss of kidney function and positioned RTX, an anti-CD20 B-cell-depleting monoclonal antibody, as a first-line option for patients at moderate to high risk [4]. Randomized trials, including MENTOR [5] and GEMRITUX [6], established the efficacy of RTX, which induces remission in 60–80% of patients. Nonetheless, 20–40% fail to respond, and 5–28% of responders subsequently relapse [7], while the individual clinical course remains difficult to predict. Although anti-PLA2R1 antibody titers and proteinuria reflect humoral activity and glomerular injury in 70-80 % of patients, they only partially explain this clinical heterogeneity and frequently lag behind the underlying cellular events [8].

Beyond B-cell depletion, regulatory and innate immune compartments have been implicated in the course of pMN, although the evidence derives from small cohorts using heterogeneous phenotypic definitions [9]. Circulating CD4+CD25+FoxP3+ T cells (Tregs) are reduced in pMN compared with healthy individuals [10,11]. In patients with severe pMN treated with RTX, responders had lower baseline Treg frequencies than non-responders and an increase as early as day 8 after infusion, whereas Tregs remained unchanged in non-responders [10]. After a four-dose regimen, a Treg increase between months 6 and 12 was likewise confined to responders [12]. Data on CD19+CD5+ B cells (Bregs) are fewer and discordant. B cells with regulatory phenotypes have been reported as reduced [13], increased [14], or expanded but skewed towards pro-inflammatory cytokine production [15] in pMN. These discrepancies partly reflect the absence of a lineage-defining marker for human Bregs, which can arise at several stages of B-cell development and are defined chiefly by IL-10 competence [16]. In this context, CD5 is one surface marker associated with IL-10 production by B cells [17]. Similarly, CD25 and FoxP3 are also expressed by activated, non-suppressive CD4+ T cells so that additional markers such as CD127 are needed to delineate Tregs precisely [18,19,20]. The innate compartment has received less attention, although a composite inflammatory index, the systemic inflammation response index (SIRI), was recently associated with RTX response [21]. Because these compartments evolve dynamically after infusion, a single cross-sectional measurement is unlikely to capture the immunological trajectory that determines outcome. To date, immune monitoring in pMN has focused almost exclusively on the B-cell compartment, with recent work establishing that on-treatment B-cell indices predict remission [21]. Whether the T-cell and innate compartments carry information independent of B-cell kinetics has not been addressed. We therefore hypothesized that longitudinal peripheral immunophenotyping, integrating adaptive and innate readouts across the full RTX treatment arc, could identify cellular biomarkers that associate with RTX response.

## 2. Results

### 2.1. Baseline Characteristics and Outcomes

Thirty-five patients with primary MN (51.4% anti-PLA2R antibody positive) were treated with the standard two-dose RTX regimen. Twenty-three patients (65.7%) achieved partial or complete remission (responders), within a mean of 7.48 ± 2.4 months. Of the 23 responders, 10 (43.5%) achieved complete remission and received no further RTX treatment, while 13/23 (56.5%) achieved only partial remission and required RTX re-treatment. Twelve patients (34%) did not respond. Responders and non-responders were comparable in age (62.65 ± 13.8 vs. 58 ± 14.3 years, *p* = 0.357), sex distribution (male 56.5% vs. 75%, *p* = 0.283) and in the prevalence of diabetes mellitus (26.1% vs. 25%, *p* = 0.944), hypertension (69.6% vs. 58.3%, *p* = 0.506), cardiovascular disease (34.8% vs. 16.7%, *p* = 0.260) and microscopic hematuria (39.1% vs. 50%, *p* = 0.537). Anti-PLA2R antibody positivity did not differ (52.2% vs. 50%, *p* = 0.903), nor did serum albumin (23.1 ± 2.8 vs. 22.7 ± 3.1 g/L, *p* = 0.7), C-reactive protein (4.6 [3,4,5,6] vs. 5.7 [4.2–6.4] mg/L, *p* = 0.149) or any lipid parameter (Supplementary Table S1). Non-responders differed in three aspects, all in the direction of more severe disease at presentation: higher body weight (86.33 ± 12.8 vs. 74.5 ± 13.8 kg, *p* = 0.019), lower eGFR (51 ± 19.3 vs. 65.9 ± 19.2 mL/min per 1.73 m^2^, *p* = 0.035) and higher proteinuria (UPCR 14.6 ± 4.4 vs. 10.4 ± 3.06 g/g, *p* = 0.009) (Table 1, full data in Supplementary Table S1). Seven patients (20%) from the total cohort had relapsing pMN and had received prior immunosuppression (cyclosporine in three and a modified Ponticelli regimen in four) discontinued in every case at least 12 months before baseline in accordance with the exclusion criteria. The proportion did not differ between groups (17% vs. 25%, *p* = 0.593).

### 2.2. Baseline Immunophenotype

At baseline, future non-responders had fewer CD19+CD5+ B cells (0.32 versus 0.52%, *p* = 0.005), a higher CD3+CD4+ proportion (38.6 versus 32.6%, *p* = 0.038) and more naïve CD4+CD45RA+ cells (27 versus 20.04%, *p* = 0.01), and the SIRI tended to be higher (1.82 versus 1.58, *p* = 0.052). None of these differences remained significant after Benjamini–Hochberg (FDR) correction. Total B-cell, memory B-cell, CD4+CD25+FoxP3+ T-cell and monocyte proportions did not differ (Table 2 full data in Supplementary Table S2). All differences at months 3 and 6 reported below remained significant after the FDR correction.

### 2.3. Immune Profile at Month 3

By month 3, the compartments differed markedly (Table 2). Responders showed deeper B-cell depletion (residual CD3−CD19+ 0.44 versus 1.015%, *p* < 0.001) and higher CD4+CD25+FoxP3+ T cells (4.6 versus 3.19%, *p* < 0.001). Within the innate compartment, responders had lower intermediate CD14++CD16+ monocytes (6.75 versus 8.96%, *p* < 0.001) and lower SIRI (1.32 versus 1.48, *p* < 0.001).

### 2.4. Immune Profile at Month 6

**Figure 1:**
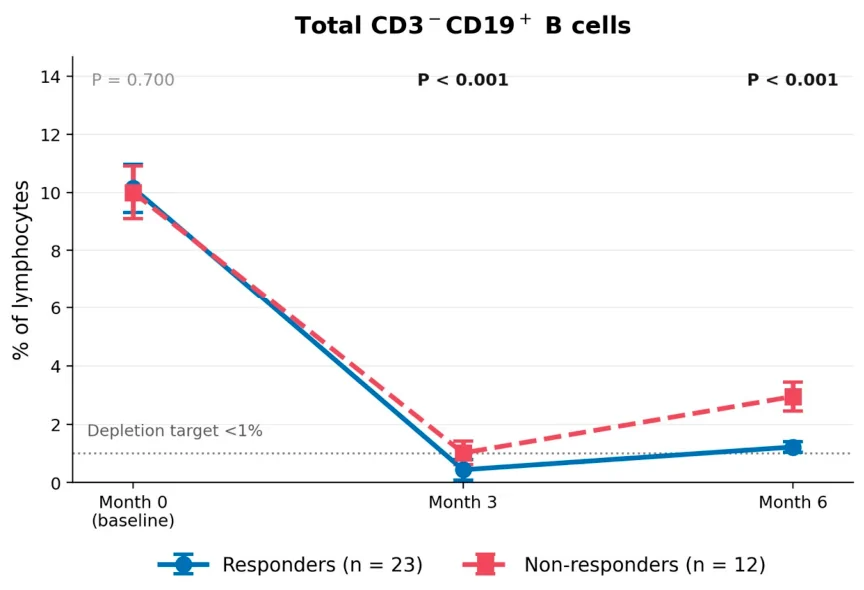
Total CD3-CD19+ in months 0, 3 and 6 in responders vs. non-responders.

**Figure 2:**
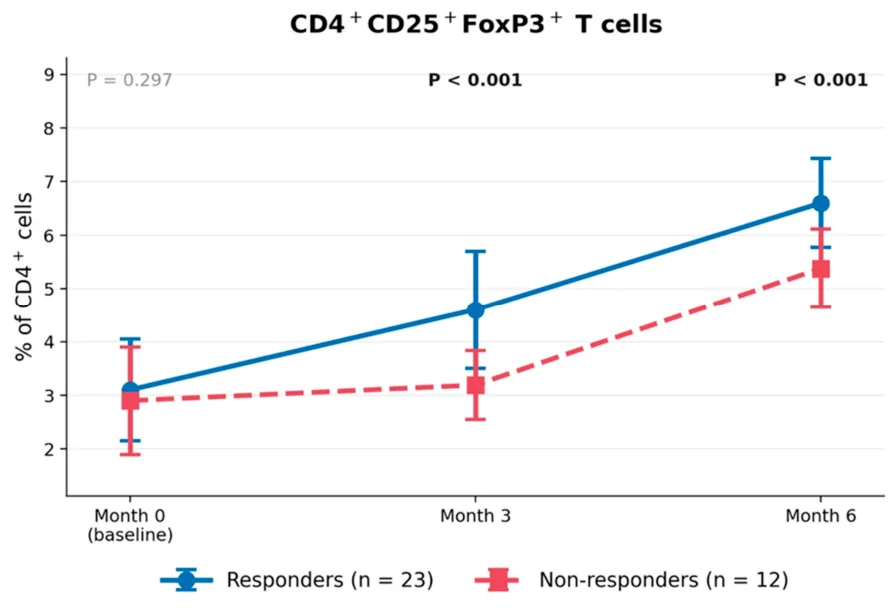
CD4+CD25+FoxP3+ at months 0, 3 and 6 in responders vs. non-responders.

**Figure 3:**
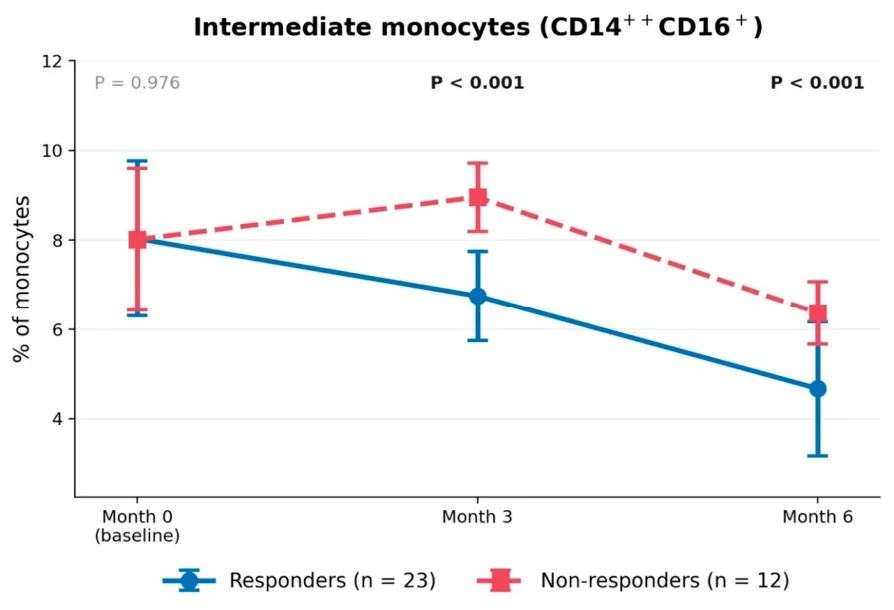
CD14++CD16+ intermediate at months 0, 3 and 6 in responders vs. non-responders.

By month 6, responders continued to show durable B-cell depletion (residual CD3−CD19+ 1.22 versus 2.95%, *p* < 0.001) and higher CD4+CD25+FoxP3+T cells (6.6 versus 5.38%, *p* < 0.001). Within the innate compartment, responders continued to have lower intermediate CD14++CD16+ monocytes (4.67 versus 6.37%, *p* < 0.001), while the SIRI no longer differed (*p* = 0.78). At months 3 and 6, the profound reduction in total CD3−CD19+ B cells left too few B-cell events to resolve switched-memory, non-switched-memory, naïve and CD19+CD5+ B-cell subpopulations reliably by flow cytometry. These subsets are therefore not reported at those time points. Month-6 immunophenotyping was performed before the third RTX dose in partial responders. The total CD19+, CD4+CD25+FoxP3+ and CD14++CD16+ trajectories at months 0, 3 and 6 are presented in Figure 1, Figure 2 and Figure 3.

### 2.5. Longitudinal Trajectories

In linear mixed-effects models across months 0, 3 and 6, all four parameters changed significantly over time (main effect of time, all *p* < 0.001), and all differed between responders and non-responders (main effect of response status: total CD3−CD19+ B cells F (1,33) = 42.85, *p* < 0.001; CD4+CD25+FoxP3+ T cells F (1,33) = 25.73, *p* < 0.001; intermediate CD14++CD16+ monocytes F (1,33) = 12.13, *p* = 0.001; SIRI F (1,33) = 5.70, *p* = 0.023). The shape of the trajectory differed between groups only for total CD3−CD19+ B cells (group × time interaction F (2,33) = 26.05, *p* < 0.001), which were indistinguishable at baseline and separated thereafter, with responders achieving deeper depletion at month 3 and less repopulation by month 6. For CD4+CD25+FoxP3+ T cells (F (2,33) = 1.55, *p* = 0.227), intermediate monocytes (F (2,33) = 2.14, *p* = 0.134) and the SIRI (F (2,33) = 0.70, *p* = 0.505), the group × time interaction did not reach significance. Given the limited power of interaction tests at this sample size, this does not establish parallel trajectories. CD4+CD25+FoxP3+ T cells and intermediate monocytes did not differ at baseline but differed at months 3 and 6, and the SIRI differed at month 3 but not at month 6 (Table 2). The addition of concurrent proteinuria as a time-varying covariate did not materially alter the time effect for CD4+CD25+FoxP3+ T cells, intermediate CD14++CD16+ monocytes or the SIRI (within-patient ln-UPCR, all *p* > 0.05).

### 2.6. Factors Associated with Remission

**Figure 4:**
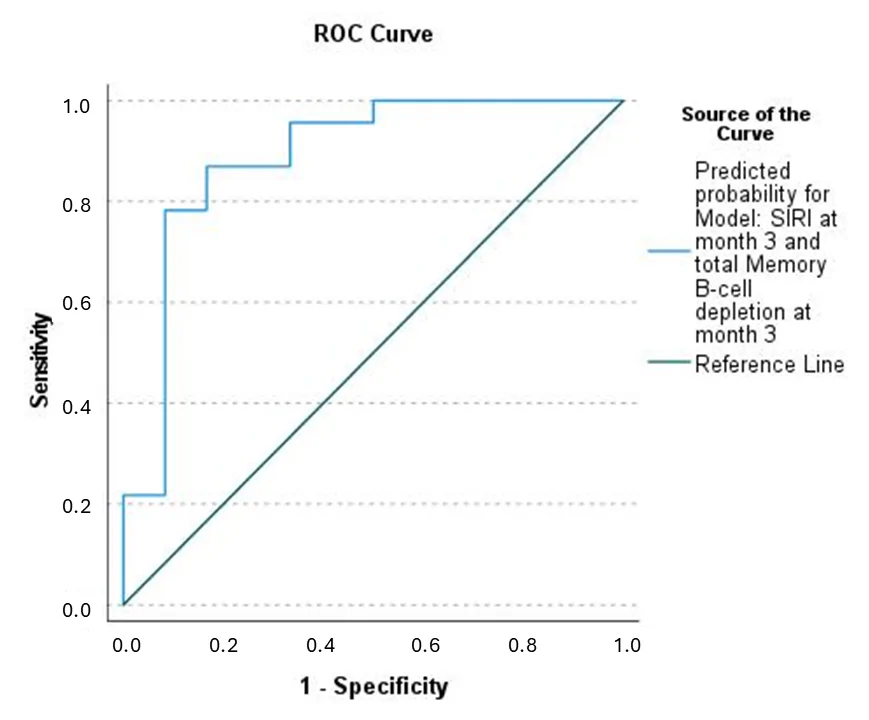
Hypothesis-generating receiver-operating-characteristic curve for the model combining the SIRI and depletion of total CD3−CD19+ B cells at month 3. Apparent, in-sample area under the curve 0.87 (95% CI 0.76–0.98); sensitivity 87%, specificity 83%. The cut-off and the model were derived and evaluated in the same 35 patients. The diagonal reference line denotes no discrimination.

**Figure 5:**
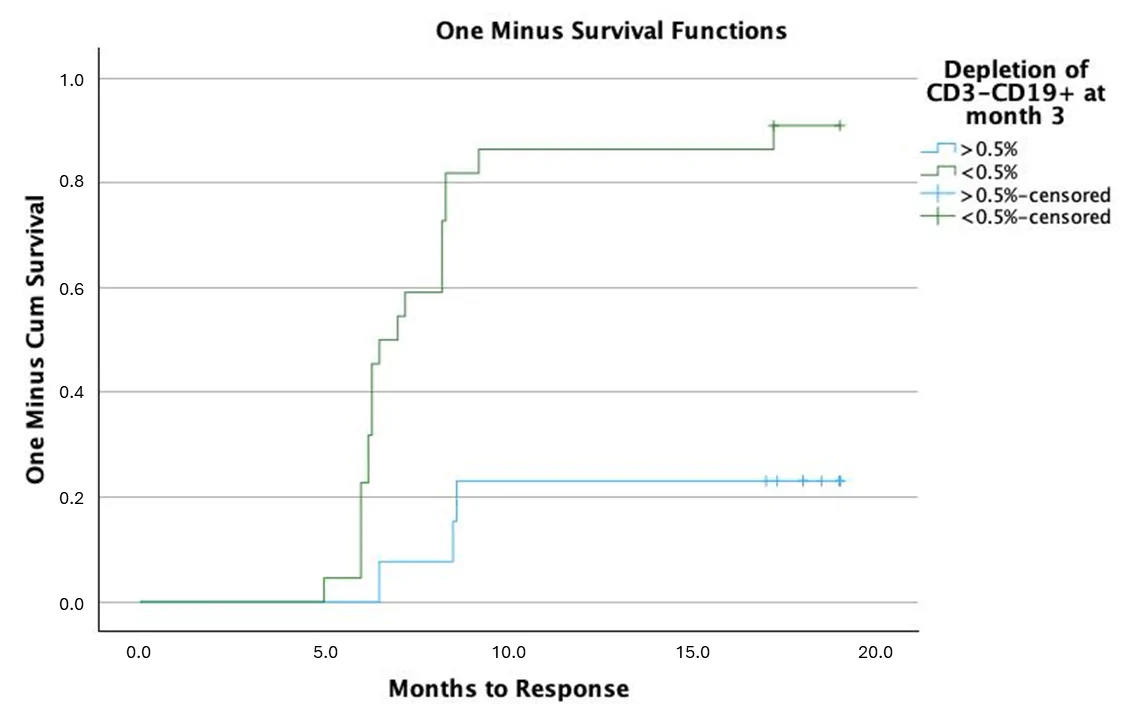
Conditional landmark Kaplan–Meier curves (one minus survival) for time to remission measured from the month-3 landmark, stratified by depletion of total CD3−CD19+ B cells to below 0.5% at month 3 (yes versus no). Log-rank *p* < 0.001. No patient achieved remission prior to month 3.

On univariate logistic regression, remission (complete or partial) was associated with depletion of total CD3−CD19+ B cells at month 3 (defined as CD3−CD19+ < 0.5%, odds ratio (OR) 5.91, 95% CI 1.12–31.2, *p* = 0.036) and a lower SIRI at month 3 (OR 0.44, 95% CI 0.22–0.92, *p* = 0.029), together with higher CD4+CD25+FoxP3+ T cells at month 3 (OR 3.43, 95% CI 1.34–8.8, *p* = 0.01) and lower intermediate monocytes at month 3 (OR 0.28, 95% CI 0.11–0.74, *p* = 0.008) (Table 3). Anti-PLA2R antibody at baseline, as a nominal variable or as titer, was not significant. In the multivariate model, month-3 total CD3−CD19+ depletion (OR 5.8, 95% CI 1.1–31.1, *p* = 0.039) and a lower month-3 SIRI (OR 0.44, 95% CI 0.22–0.91, *p* = 0.026) remained associated with remission. In a hypothesis-generating analysis, a model combining these two month-3 variables had an apparent, in-sample area under the curve of 0.87 (95% CI 0.76–0.98; sensitivity 87%, specificity 83%; Figure 4). Because the cut-off and the model were derived and evaluated in the same 35 patients, this estimate is likely to be optimistic. Complete depletion of total CD3−CD19+ B cells at month 3 also stratified the time to remission (log-rank *p* < 0.001, Figure 5).

### 2.7. Sensitivity Analysis

Three post hoc analyses were performed to examine the two variables retained in the multivariate model. First, because non-responders entered the study with a lower eGFR, and both intermediate monocytes and the SIRI rise as kidney function declines, baseline eGFR was forced into the multivariate model. Baseline eGFR (OR = 1.09, 95% CI = 0.95–1.25, *p* = 0.22) as well as baseline UPCR (OR = 0.29, 95% CI = 0.068–1.21, *p* = 0.091) and baseline body weight (OR = 0.92, 95% CI = 0.85–1.002, *p* = 0.057) were added to the multivariate model separately, with none reaching significance. Month-3 total CD3−CD19+ depletion and month-3 SIRI remained associated with remission in all adjusted models. However, with 12 non-response events, each covariate-adjusted model had four events per variable and was prone to sparse-data bias. Therefore, these estimates should be interpreted with caution. Second, excluding the seven patients with relapsing pMN and prior immunosuppression did not materially alter either estimate, although precision was reduced and the lower confidence limit approached unity (CD3−CD19+ depletion OR = 6.2, 95% CI = 1.01–38.1, *p* = 0.049, SIRI OR 0.47, CI = 0.24–0.93, *p* = 0.029, n = 28). Third, percentile bootstrap resampling with 1000 samples yielded a CI= 4.1–601 for CD3−CD19+ depletion (bootstrap *p* = 0.001) and a CI = 0.11–0.99 for the SIRI (bootstrap *p* = 0.042). The width of these intervals reflects the small number of non-response events.

### 2.8. The Relapse Signal: A Hypothesis

Six (26%) out of the 23 responders relapsed within a mean of 20.2 ± 6.3 months from baseline. Relapse was heralded by re-expansion of class-switched memory B cells (CD19+CD27+IgD−; 17.7 versus 14.25%, *p*< 0.001), accompanied by a higher SIRI (1.72 versus 1.17, *p* < 0.001) and higher intermediate CD14++CD16+ monocytes (7.26 versus 4.9%, *p* = 0.043) at month 12 in relapsing responders. Of note, only 10/23 (43.5%) patients achieved complete remission through the first RTX induction, while the remaining 13 (56.5%) required an additional RTX course in month 6. Given only six relapse events, and the confounding factor of the third RTX dose, no univariate or multivariate logistic regression model was fitted, and these analyses are considered exploratory.

## 3. Discussion

In this prospective single-center cohort, longitudinal peripheral immunophenotyping across the full RTX treatment arc identified candidate cellular biomarkers associated with RTX response. At baseline, non-responders had fewer CD19+CD5+ B cells, although no baseline difference survived correction for multiple testing. Deeper B-cell depletion, higher CD4+CD25+FoxP3+ T cells, lower intermediate monocytes and a lower SIRI at month 3 were associated with remission on a univariate level. In mixed-effects models, the group × time interaction was significant only for the B-cell compartment (*p* < 0.001). For CD4+CD25+FoxP3+ T cells, intermediate monocytes and the SIRI, it did not reach significance, which, given the limited power, does not exclude divergence after treatment.

Before any exposure to RTX, patients destined not to respond had nominally fewer CD19+CD5+ B cells, a higher CD3+CD4+ proportion and a larger naïve CD4+CD45RA+ pool, and the SIRI tended to be higher, but none of these differences survived correction for multiple testing. The CD19+CD5+ finding warrants particular caution: this subset was measured at low frequencies without fluorescence-minus-one controls, and because Breg function was not assessed, it cannot be equated with functional Bregs. Whether a pre-existing deficit in B-cell-mediated regulation marks patients in whom depletion alone will not suffice therefore remains a hypothesis for larger studies.

Rosenzwajg et al. found that responders had lower baseline Treg frequencies than non-responders, followed by an early post-infusion rise confined to responders [10]. In our cohort, baseline CD4+CD25+FoxP3+ T cells did not differ, and the only nominal baseline regulatory difference, fewer CD19+CD5+ B cells, was found in non-responders. If confirmed, a baseline regulatory deficit would thus be associated with response in their cohort but with non-response in ours, a discrepancy that may reflect the compartment studied (T versus B cells), phenotypic definitions and disease severity. Extending their observation of an early post-infusion rise, we found higher CD4+CD25+FoxP3+ T cells in responders at months 3 and 6, a pattern compatible with, but not demonstrating, recovery of regulatory function.

A distinctive aspect of our findings is the contribution of the innate compartment. Intermediate CD14++CD16+ monocytes, a well-recognized proinflammatory subset in the standard three-tier monocyte nomenclature [22], were lower in responders at month 3 and 6, whereas the SIRI, a composite of neutrophil, monocyte and lymphocyte counts, was associated with the outcome at month 3. Together these suggest that resolution of innate inflammation parallels humoral silencing in successful RTX therapy and that simple, widely available indices such as the SIRI may serve as accessible surrogates. An important caveat applies to this innate signal. Intermediate CD14++CD16+ monocytes expand as kidney function declines and are independently associated with cardiovascular outcome in non-dialysis CKD [23], and the SIRI is similarly sensitive to the inflammatory milieu of uremia. Since our non-responders entered the study with a lower eGFR, a contribution of baseline kidney function to the observed monocyte and SIRI differences cannot be excluded, despite adjustment for baseline eGFR.

The strongest association with remission was the depth of B-cell depletion at month 3. This is mechanistically coherent: RTX exerts its effect by eliminating the CD20+ B-cell pool from which short-lived, autoantibody-secreting plasmablasts arise, and more complete early depletion plausibly reflects both adequate drug exposure and a less entrenched autoreactive compartment. Consistent with this, profound month-3 depletion (total CD3−CD19+ < 0.5%) was associated with remission, whereas baseline anti-PLA2R antibody status and titer were not. This is concordant with the accumulated experience of B-cell-guided RTX therapy in pMN. Cravedi et al. showed that titrating RTX to circulating CD20+ B cells achieved persistent depletion and remission rates equivalent to the standard four-dose regimen [24], and in the extended series of 100 consecutive patients reported by Ruggenenti et al., 65 achieved complete or partial remission with a median time to remission of 7.1 months [25]—closely comparable to the 65.7% remission rate and mean time to response of 7.48 months observed here. Duan et al. subsequently showed that on-treatment B-cell indices combined with the SIRI improve prediction of remission [21]. Our data add that the discriminating measurement can be made as early as month 3 and that a threshold of <0.5% total CD3−CD19+ B cells may be sufficient. This threshold, however, was derived in our cohort and therefore requires external validation.

Our responder and non-responder groups were not clinically comparable at baseline: non-responders had higher body weight, lower eGFR and higher proteinuria. This differs from the cohorts against which our findings are most naturally compared, in which groups were balanced by design [24] or differed only immunologically [10]. The imbalance affects interpretation in two ways. First, as noted above, intermediate monocytes and the SIRI are sensitive to kidney function and systemic inflammation, so their association with response may partly reflect baseline disease severity. Some observations argue against a purely severity-driven explanation for the monocyte signal: intermediate monocytes did not differ at baseline (*p* = 0.976) when the eGFR difference was already present and diverged only after treatment, and baseline C-reactive protein was similar (*p* = 0.149). The SIRI, however, already tended to be higher at baseline and is more exposed to this concern. Second, baseline severity may influence drug exposure. With a fixed 2 × 1 g regimen, heavier patients receive a lower weight-normalized dose, and heavy proteinuria is associated with lower serum RTX concentrations [26]. Reduced RTX bioavailability is a recognized mechanism of treatment resistance in pMN [27]. The less complete B-cell depletion in non-responders may therefore partly reflect lower RTX exposure rather than an intrinsic immunological difference. Our sensitivity analyses adjusted for baseline eGFR, body weight, UPCR and, in the mixed-effects models, for concurrent proteinuria. With only 12 non-response events, residual confounding by unmeasured factors cannot be excluded, and serum RTX levels were not measured. The associations we report are therefore best interpreted as markers of the trajectory towards remission rather than as independent determinants of response.

Clinically, these observations point towards a practical monitoring strategy. If validated prospectively, a month-3 assessment of B-cell depletion, possibly complemented by the SIRI, could identify patients at risk of non-response early enough to consider treatment intensification. Whether acting on such a signal, for example, by intensifying treatment, improves outcomes was not evaluated in this study and would require a prospective biomarker-guided trial. Because these measures rely on standard flow cytometry and routine blood counts, they would in principle be deployable beyond specialized centers.

Our study has limitations. It is a single-center study of modest size. The flow-cytometry panel did not resolve plasmablasts or long-lived plasma cells, nor functional regulatory B-cell or T-follicular-helper subsets. The relatively low proportion of anti-PLA2R-positive patients (51.4%) compared with the 70–80% reported in the literature may limit generalizability. Profound B-cell depletion at months 3 and 6 precluded reliable resolution of B-cell subpopulations at those timepoints. Fluorescence-minus-one and isotype controls were not used, so gate placement for low-frequency populations such as CD19+CD5+ B cells and FoxP3+ cells relied on visual inspection. All prediction analyses in this study are exploratory. Cut-offs were derived by the Youden index, and the multivariate model was fitted and evaluated within the same 35 patients. Data-driven cut-offs overestimate sensitivity and specificity in samples of this size [28], and the area under the curve of 0.87 is an in-sample estimate that is likely to be optimistic. With 12 non-response events, the multivariate model was selected from four candidate variables and retained two (events per variable: three at entry and six in the final model), well below the numbers recommended for developing prediction models [29]. The bootstrap resampling assessed the stability of the regression coefficients, not the model’s performance, and does not replace validation in an independent cohort. Calibration was not assessed, because in-sample calibration is uninformative and an optimism-corrected estimate would be unstable with 12 non-response events. Finally, interaction tests in the mixed-effects models had limited power at this sample size. Prospective, multicenter validation with standardized panels is required before these readouts can inform treatment.

## 4. Materials and Methods

### 4.1. Patients and Inclusion Criteria

We prospectively included patients with primary MN, either de novo or relapsing, diagnosed with kidney biopsy or by anti-PLA2R antibody positivity, who underwent RTX treatment at the University Hospital of Ioannina between June 2023 and November 2025. Baseline was defined as the time of the first RTX administration. All patients received the standard RTX protocol: two 1 g infusions given two weeks apart. At the 6-month follow-up, a second course of RTX was administered only to patients who had achieved partial remission (per KDIGO 2021 [4]). Patients who achieved complete remission received no further treatment, and patients achieving neither complete nor partial remission were censored at the date of last follow-up and received no further RTX. All patients were treated with the maximum tolerated supportive therapy with angiotensin receptor blockers (ARBs) and sodium glucose cotransporter-2 inhibitors (SGLT2-i).

### 4.2. Data Acquisition

Baseline assessment included demographic information, clinical symptoms, laboratory data and kidney pathology. Serum creatinine, estimated glomerular filtration rate (eGFR; calculated with the 2021 CKD-EPI creatinine equation), albumin, urine protein-to-creatinine ratio (UPCR), urine albumin-to-creatinine ratio (UACR), lipid parameters and inflammatory markers (erythrocyte sedimentation rate, C-reactive protein) were measured monthly after treatment to assess response. Anti-PLA2R antibody levels and the systemic inflammation response index (SIRI) were measured at baseline and at months 3, 6 and 12.

### 4.3. Exclusion Criteria

The following conditions excluded patients from the study:

(1) Patients with secondary causes of membranous nephropathy;
(2) Patients with active malignancy;
(3) Patients with severe heart failure (NYHA class IV) or hepatic failure;
(4) Patients with active infection or inflammation;
(5) Patients receiving, or having received within the preceding 12 months, immunomodulatory agents for primary membranous nephropathy or other conditions.

### 4.4. Flow Cytometry

Peripheral-blood immune-cell subsets were analyzed by flow cytometry in a 100 µL whole-blood assay within 8 h of sampling. Whole blood (2 mL) was collected into ethylenediaminetetraacetic acid tubes under standardized conditions. Flow cytometry was performed at baseline and at months 3, 6 and 12. Immunophenotypic profiling at month 0 was performed prior to the administration of the 1st RTX dose, and prior to the administration of the 3rd RTX dose at month 6 in patients with partial remission. The following conjugated monoclonal antibodies were used: CD45, CD14, CD16, HLA-DR, CD4, CD8, CD56, CD3, CD19, CD25, CD27, CD5, IgD, CD45RA, CD45RO and FoxP3. Samples were acquired on a flow cytometer (FACSCalibur; Becton Dickinson, Franklin Lakes, NJ, USA) and analyzed with BD CellQuest Pro v.5.1 and BD FACSDiva software v.9.0. Intracellular FoxP3 staining was performed after fixation and permeabilization using the eBioscience™ Foxp3/Transcription Factor Staining Buffer Set (Thermo Fisher Scientific, Waltham, MA, USA), according to the manufacturer’s instructions. Cell populations were quantified at baseline and at months 3 and 6 for the response analysis and at month 12 for the relapse analysis. They are reported as percentages, with absolute lymphocyte and monocyte counts retained for reference. Supplementary Table S5 lists the fluorochrome assigned to each marker in each tube. Fluorescence compensation was established using standard methods with BD CompBeads (cat no. 552843). Monocytes were gated first and then divided by CD14 and CD16 expression into classical, intermediate and non-classical subsets [22]. For T-cell phenotyping, lymphocytes were gated first, followed by identification of CD4^+^ T cells. CD4^+^CD25^+^FoxP3^+^ T cells were identified within the CD4+ gate. For B-cell phenotyping, lymphocytes were gated first, followed by identification of CD19^+^ B cells. CD19^+^CD5^+^ B cells were assessed using a separate panel. B-cell subsets were further characterized according to CD27 and IgD expression. FMO and isotype controls were not used in this study. Gates were positioned by visual inspection of fluorescence distributions. The B-cell compartment comprised total CD3−CD19+ (% of lymphocytes) and, as % of CD19+, CD19+CD27+IgD+ (non-switched memory), CD19+CD27+IgD− (class-switched memory), CD19+CD27−IgD+ (naïve) and CD19+CD5+ B cells. The T-cell compartment comprised CD3+, CD3+CD4+ and CD3+CD8+ (% of lymphocytes), the CD4/CD8 ratio and, as % of CD4+, CD4+CD25+FoxP3+ T cells, CD4+CD45RA+ (naïve), CD4+CD45RO+ (memory) and CD45RA+RO+ (transitional). Monocyte subsets (% of monocytes) were CD14++CD16− (classical), CD14++CD16+ (intermediate), CD14+CD16++ (non-classical) and CD14+HLA-DR+ (activated). Natural killer cells (CD16+CD56+) were expressed as % of total lymphocytes. The SIRI was computed as (neutrophil count × monocyte count)/lymphocyte count. Flow-cytometry analyses were performed by operators blinded to clinical outcomes. Repeatability was assessed by re-acquiring the same stained sample (intra-assay) and by repeating the staining and acquisition procedures on the same blood sample (inter-assay).

### 4.5. Lower Limit of Quantification and Depletion Criteria

For each tube, 20,000 events were acquired in the lymphocyte gate. The lower limit of quantification (LLOQ) for the CD3−CD19+ gate, defined as 25 gated events (coefficient of variation ≤ 20%), was 0.125% of lymphocytes. Depletion was defined as CD3−CD19+ < 0.5% of lymphocytes, corresponding to 100 gated events (coefficient of variation 10%), four times the LLOQ. Values below the LLOQ were classified as depleted. Absolute counts were obtained by dual-platform calculation from the hematology analyzer lymphocyte count. The cells/μL criterion was not used in the analysis. At month 3, every patient with <5 cells/μL also had <0.5% of lymphocytes. In total, 2 of 35 patients had <0.5% but ≥5 cells/μL and were also classified as depleted by the depletion criterion.

### 4.6. Phenotypic Definitions and Their Limitations

CD19+CD5+ B cells were enumerated as a subset enriched for IL-10–competent B cells [13,17]. Because CD1d, CD24, CD38 and intracellular IL-10 were not assessed, this gate also contains CD5+ B cells without regulatory function and is not equivalent to functionally defined Bregs [16]. CD4+CD25+FoxP3+ T cells were enumerated as a phenotypic surrogate of Tregs. Without CD127 co-staining, this population may include recently activated conventional CD4+ T cells that express CD25 and FoxP3 without suppressive function [18,19,20]. No functional assays were performed, and both populations are therefore designated by their phenotype throughout.

### 4.7. Endpoints

The primary endpoints were remission after RTX, comprising complete remission (UPCR < 0.3 g/g and serum albumin > 35 g/L) or partial remission (≥50% reduction in proteinuria from baseline with UPCR < 3.5 g/g) and disease relapse (re-emergence of proteinuria with UPCR > 3.5 g/g after complete remission or increase of UPCR > 50% from the nadir after partial remission). Patients were followed to a total of 12 months to assess response to treatment. Responders were then followed to assess disease relapse.

### 4.8. Statistical Analysis

Normally distributed continuous variables are presented as mean ± standard deviation (SD) and were compared with an independent-samples *t*-test, using Welch’s correction when Levene’s test indicated unequal variances. Non-normally distributed variables are presented as median (interquartile range) and were compared with the Mann–Whitney *U*-test. Categorical variables were compared with the chi-squared test. Given the exploratory design, nominal *p*-values are reported. In addition, between-group comparisons were corrected for multiple testing within each time point using the Benjamini–Hochberg (FDR) procedure, with an FDR-adjusted *p*-value (q) < 0.05 considered significant. *p*-values from the regression analyses are nominal and should be interpreted as hypothesis-generating. Where appropriate, receiver-operating-characteristic (ROC) analysis with the Youden index defined optimal cut-offs for continuous variables. Univariate logistic regression identified variables associated with the endpoints (*p* < 0.05), and significant variables entered multivariate logistic regression using a backward conditional method. Model discrimination was assessed by ROC analysis as an apparent, hypothesis-generating estimate. The 95% CI of the area under the curve is the nonparametric asymptotic interval computed by SPSS v.31.0.0.0. To assess the stability of the regression coefficients, percentile bootstrap resampling was performed with 1000 samples; bootstrap 95% CIs and *p*-values are reported alongside the original estimates for the variables retained in the multivariate model. Because depletion status was determined at month 3, time-to-remission analyses stratified by on-treatment variables were performed as conditional landmark analyses to avoid guarantee-time (immortal time) bias [30]. Baseline clinical variables that differed between groups (body weight, eGFR and UPCR) were not included among the candidate variables because, with 12 non-response events, the model could not reliably accommodate additional covariates [29]. The influence of these 3 variables was instead examined in post hoc sensitivity analyses, by forcing baseline eGFR, UPCR and body weight into the final model and by adding concurrent UPCR as a time-varying covariate to the linear mixed-effects models. The landmark was set at the timepoint at which the stratifying variable was measured; patients reaching the endpoint or censored before the landmark were excluded, and follow-up was measured from the landmark rather than from baseline. Landmarks were month 3 for total CD3−CD19+ depletion, CD4+CD25+FoxP3+ T cells, intermediate monocytes and the SIRI. Analyses were performed with SPSS version 31.0.

## 5. Conclusions

In this exploratory prospective cohort, deeper B-cell depletion and a lower SIRI at month 3 were associated with remission after RTX in pMN, alongside differences in CD4+CD25+FoxP3+ T cells and intermediate monocytes. These associations are best interpreted as markers of the trajectory towards remission rather than as independent determinants of response. Validation in larger, multicenter cohorts with standardized panels is required before clinical use.

## Figures and Tables

**Table 1:** Baseline clinical characteristics of responders and non-responders. Values are mean ± SD or median (interquartile range) unless otherwise stated. eGFR, estimated glomerular filtration rate; PLA2R, phospholipase A2 receptor; SD, standard deviation; UACR, urine albumin-to-creatinine ratio; UPCR, urine protein-to-creatinine ratio. The full panel is presented in Supplementary Table S1.

Characteristic	All (n = 35)	Responders (n = 23)	Non-Responders (n = 12)	*p*
Age (years)	61.06 ± 13.9	62.65 ± 13.8	58 ± 14.3	0.357
Male, n (%)	22 (62.9)	13 (56.5)	9 (75)	0.283
Diabetes mellitus, n (%)	9 (25.7)	6 (26.1)	3 (25)	0.944
Body weight (kg)	78.6 ± 14.4	74.5 ± 13.8	86.33 ± 12.8	0.019
Hypertension, n (%)	23 (65.7)	16 (69.6)	7 (58.3)	0.506
Cardiovascular disease, n (%)	10 (28.6)	8 (34.8)	2 (16.7)	0.260
Microscopic hematuria, n (%)	15 (42.9)	9 (39.1)	6 (50)	0.537
Anti-PLA2R antibody positive, n (%)	18 (51.4)	12 (52.2)	6 (50)	0.903
Prior immunosuppression, n (%)	7 (20)	4 (17)	3 (25)	0.593
eGFR (mL/min per 1.73 m^2^)	60.8 ± 20.3	65.9 ± 19.2	51 ± 19.3	0.035
UPCR (g/g)	11.8 ± 4.05	10.4 ± 3.06	14.6 ± 4.4	0.009
UACR (g/g)	8.36 ± 2.24	7.98 ± 2.21	9.08 ± 2.2	0.178
Albumin (g/L)	22.96 ± 2.9	23.1 ± 2.8	22.7 ± 3.1	0.70
Creatinine (µmol/L)	117 ± 57	106 ± 37	138 ± 82	0.221
C-reactive protein (mg/L)	5.2 (3–6.2)	4.6 (3–6)	5.7 (4.2–6.4)	0.149
Total cholesterol (mmol/L)	8.0 ± 1.0	8.1 ± 0.9	7.9 ± 1.2	0.652

**Table 2:** Immunophenotypic parameters at baseline, month 3 and month 6 compared between responders and non-responders. Values are mean ± SD. CD19+ subsets are expressed as % of total CD19+ cells; CD4+CD25+FoxP3+ T cells and naïve CD4+ cells as % of CD4+ cells; monocyte subsets as % of monocytes; total CD3−CD19+ cells as % of lymphocytes. SD, standard deviation. Full subset data are presented in Supplementary Tables S2–S4. *p*-values are nominal; ^†^ marks differences that remained significant after Benjamini–Hochberg false discovery rate correction within each time point (q < 0.05).

Parameter	Responders (n = 23)	Non-Responders (n = 12)	*p*
Baseline (month 0)			
CD19+CD5+ B cells (%)	0.52 ± 0.21	0.32 ± 0.17	0.005
CD3+CD4+ (%)	32.6 ± 7.8	38.6 ± 7.7	0.038
Naïve CD4+CD45RA+ (%)	20.04 ± 6.28	27 ± 7.19	0.010
Systemic inflammation response index	1.58 ± 0.2	1.82 ± 0.37	0.052
Month 3			
Residual CD3−CD19+ B cells ^†^ (%)	0.44 ± 0.05	1.015 ± 0.4	<0.001
T cells CD4+CD25+FoxP3+ ^†^ (%)	4.6 ± 1.1	3.19 ± 0.64	<0.001
Intermediate CD14++CD16+ monocytes ^†^ (%)	6.75 ± 1	8.96 ± 0.76	<0.001
Systemic inflammation response index ^†^	1.32 ± 0.13	1.48 ± 0.16	<0.001
Month 6			
Residual CD3−CD19+ B cells ^†^ (%)	1.22 ± 0.19	2.95 ± 0.5	<0.001
T cells CD4+CD25+FoxP3+ ^†^ (%)	6.6 ± 0.83	5.38 ± 0.73	<0.001
Intermediate CD14++CD16+ monocytes ^†^ (%)	4.67 ± 1.5	6.37 ± 0.7	<0.001

**Table 3:** Univariate and multivariate logistic regression models for remission. Binary logistic regression with maximum-likelihood estimation; odds ratios with Wald 95% confidence intervals and Wald *p*-values. All models included 35 patients (23 remissions, 12 non-remissions). Variables with univariable *p* < 0.05 (CD3−CD19+ depletion, SIRI, CD4+CD25+FoxP3+ T cells and intermediate CD14++CD16+ monocytes) entered backward conditional selection (removal at *p* > 0.10), which retained CD3−CD19+ depletion and SIRI. Odds ratios for continuous variables are per 1% increase for cell subsets, per 0.1-unit increase for SIRI and per 10-fold increase for anti-PLA2R titer (log10-transformed). CI, confidence interval; PLA2R, phospholipase A2 receptor; SIRI, systemic inflammation response index.

Variable	Odds Ratio (95% CI)	*p*
Univariate		
Total CD3−CD19+ depletion at month 3 (<0.5% vs. ≥0.5%)	5.91 (1.12–31.2)	0.036
Systemic inflammation response index at month 3 (per 0.1-unit increase)	0.44 (0.22–0.92)	0.029
CD4+CD25+FoxP3+ T cells at month 3 (per 1% increase)	3.43 (1.34–8.8)	0.01
Intermediate CD14++CD16+ monocytes at month 3 (per 1% increase)	0.28 (0.11–0.74)	0.008
Anti-PLA2R antibody at month 0 (positive vs. negative)	1.09 (0.27–4.4)	0.9
Anti-PLA2R antibody titer at month 0 (per 10-fold increase)	2.4 (0.72–8.05)	0.16
Multivariate		
Total CD3−CD19+ depletion at month 3 (<0.5% vs. ≥0.5%)	5.8 (1.1–31.1)	0.039
Systemic inflammation response index at month 3 (per 0.1-unit increase)	0.44 (0.22–0.91)	0.026

## Data Availability

The data that support the findings of this study are available from the corresponding author upon reasonable request.

## Supplementary Materials

The following supporting information can be downloaded at: https://www.mdpi.com/article/10.3390/ijms27219956/s1.
