# Supplementary material for: Longitudinal Peripheral Immunophenotyping and Rituximab Response in Primary Membranous Nephropathy: A Prospective Cohort Study
Source: Int J Mol Sci. 2026 Nov 6;27(21):9956. doi: 10.3390/ijms27219956 (PMC13763994; doi:10.3390/ijms27219956)
Supplement: Supplementary file 1 [file ijms-27-09956-s001.zip › ijms-4612916-supplementary.pdf]

SUPPLEMENTARY MATERIAL

Peripheral immunophenotyping identifies predictors of rituximab response in primary membranous nephropathy

Supplementary Tables

Table S1. Baseline clinical characteristics.

Characteristic	All (N=35)	Non-resp. (N=12)	Resp. (N=23)	P
**Age (years)**	61.06 ± 13.9	58 ± 14.3	62.65 ± 13.8	0.357
**Male, n (%)**	22 (62.9)	9 (75)	13 (56.5)	0.283
**Diabetes mellitus, n (%)**	9 (25.7)	3 (25)	6 (26.1)	0.944
**Body weight (kg)**	78.6 ± 14.4	86.33 ± 12.8	74.5 ± 13.8	**0.019**
**Hypertension, n (%)**	23 (65.7)	7 (58.3)	16 (69.6)	0.506
**Cardiovascular disease, n (%)**	10 (28.6)	2 (16.7)	8 (34.8)	0.26
**Microscopic haematuria, n (%)**	15 (42.9)	6 (50)	9 (39.1)	0.537
**Anti-PLA2R Ab positive, n (%)**	18 (51.4)	6 (50)	12 (52.2)	0.903
**Prior immunosuppression, n (%)**	7 (20)	3 (25)	4 (17)	0.593
**eGFR (mL/min/1.73 m²)**	60.8 ± 20.3	51 ± 19.2	65.9 ± 19.3	**0.035**
**UPCR (g/g)**	11.8 ± 4.05	14.6 ± 4.4	10.4 ± 3.06	**0.009**
**UACR (g/g)**	8.38 ± 2.24	9.08 ± 2.2	7.98 ± 2.21	0.178
**Albumin (g/dL)**	2.28 ± 0.29	2.27 ± 0.31	2.31 ± 0.28	0.70
**Haemoglobin (g/dL)**	13.9 (12.8–15.1)	13.9 (11.6–14.8)	13.9 (12.8–15.8)	0.251
**Platelets (×10³/µL)**	261 ± 75	286 ± 85	248 ± 67	0.194
**Creatinine (mg/dL)**	1.32 ± 0.65	1.56 ± 0.93	1.20 ± 0.42	0.221
**CRP (mg/L)**	5.2 (3–6.2)	5.7 (4.2–6.4)	4.6 (3–6)	0.149
**Ferritin (ng/mL)**	150 (100–200)	143 (89–176)	155 (106–209)	0.137
**Total cholesterol (mg/dL)**	310 ± 38	306.2 ± 45.6	313.1 ± 35.6	0.652
**Triglycerides (mg/dL)**	189 (168–243)	172 (165–240)	193 (173–251)	0.706

Characteristic	All (N=35)	Non-resp. (N=12)	Resp. (N=23)	P
**LDL cholesterol (mg/dL)**	199.3 ± 33.9	199.4 ± 37.6	199.3 ± 32.7	0.95
**HDL cholesterol (mg/dL)**	44.3 ± 10.3	48.2 ± 11.6	42.2 ± 9.13	0.136
**HbA1c (%)**	5.2 (5–5.4)	5 (4.9–5.3)	5 (4.8–5.2)	0.156
**ApoA1 (mg/dL)**	177.7 ± 40	163.4 ± 38.2	185.1 ± 39.7	0.132
**ApoB (mg/dL)**	89 (69–114)	86 (63–110)	94 (71–124)	0.30
**Lp(a) (mg/dL)**	32 (22–37)	28 (19–37)	33 (25–37)	0.58

Values are mean ± SD or median (IQR). Shaded rows denote P < 0.05.

Table S2. Baseline (month 0) immunophenotypic subsets.

S2a. B-cell subsets

B-cell subset (%)	All (N=35)	Non-resp. (N=12)	Resp. (N=23)	P
**Total CD3−CD19+**	10.1 ± 0.84	10 ± 0.9	10.14 ± 0.83	0.7
**CD19+CD27+IgD+ non-switched memory B**	9.5 (8.3–10.6)	9.4 (8.3–10.1)	9.5 (8.4–10.2)	0.85
**CD19+CD27+IgD− class-switched memory B**	14.7 ± 3.11	15.03 ± 3.1	14.6 ± 3.2	0.33
**CD19+CD27−IgD+ naïve**	67.5 (65–73.8)	68 (65–74)	67.8 (66.2–74.1)	0.114
**CD19+CD5+ Bregs**	0.46± 0.13	0.32± 0.06	0.52± 0.1	**0.005**

S2b. T-cell subsets

T-cell subset (%)	All (N=35)	Non-resp. (N=12)	Resp. (N=23)	P
**CD3+**	71± 4.78	69.1± 4.7	72± 4.6	0.096
**CD3+CD4+**	34.6± 8.21	38.6± 7.7	32.6± 7.8	**0.038**
**CD3+CD8+**	20.3 (19.7–22.1)	20.5 (19.5–21.9)	20.6 (19.2–22)	0.868
**CD4/CD8 ratio**	2.2 ± 0.11	2.18 ± 0.11	2.3 ± 0.1	0.3
**Tregs CD4+CD25+FoxP3+**	3.1 (2.3–4.1)	2.9 (2.1–4.1)	3.1 (2.2–4.2)	0.297
**CD4+CD45RA+ naïve**	22.4 ± 7.32	27 ± 7.19	20.04 ± 6.28	**0.01**
**CD4+CD45RO+ memory**	30.7 ± 6.89	31.9 ± 6.97	30.1 ± 6.88	0.466
**CD45RA+**	39.6 ± 9.37	40.3 ± 10.75	39.24 ± 8.8	0.755
**CD45RO+**	41.7 ± 8.8	42.3 ± 9.5	41.4 ± 8.63	0.796
**CD45RA+RO+ transitional**	2.98 ± 1.19	3.22 ± 1.17	2.85 ± 1.22	0.397

S2c. Monocyte/NK subsets and SIRI

Parameter	All (N=35)	Non-resp. (N=12)	Resp. (N=23)	P
**WBC (/µL)**	7910 (7420–9100)	7950 (7410–9000)	7850 (7550–9150)	0.576
**Lymphocytes (%)**	21.16 ± 1.07	21.12 ± 0.96	21.18 ± 1.15	0.869
**Lymphocytes (/µL)**	1583 ± 141	1570 ± 150	1590 ± 139	0.7
**Monocytes (/µL)**	643 ± 31	641.8 ± 33.3	643.6 ± 30.8	0.88
**Monocytes (%)**	8 (7.8–8.3)	8.1 (7.8–8.2)	8.1 (7.7–8.2)	0.29
**CD14++CD16− classical (%)**	78.4 (76.8–82.5)	78.6 (77–81.9)	78.5 (77.2–82.7)	0.67
**CD14++CD16+ intermediate (%)**	8.026 ± 1.65	8.014 ± 1.58	8.032 ± 1.73	0.976
**CD14+CD16++ non-classical (%)**	5.9 (4.7–8)	6 (4.7–7.9)	5.9 (4.8–8)	0.869
**CD14+HLA-DR+ activated (%)**	87.65 ± 3.68	88.98 ± 4.53	86.98 ± 3.04	0.185
**SIRI**	1.66 ± 0.29	1.82 ± 0.37	1.58 ± 0.2	**0.052**
**NK cells CD16+CD56+ (%)**	12.1 (8–18.2)	12 (8.2–18.1)	12.2 (8.1–18.2)	0.235

Shaded rows denote P ≤ 0.05.

Table S3. Month-3 immunophenotypic subsets.

S3a. B-cell subsets

B-cell subset (%)	All (N=35)	Non-resp. (N=12)	Resp. (N=23)	P
**Total CD3−CD19+ (residual)**	0.63±0.052	1.015±0.4	0.44±0.05	**<0.001**
**Total CD3-CD19+ (absolute number), cells/µL**	6.39±3.79	10.06±2.43	4.4±2.83	**<0.001**
**CD19+CD27+IgD+ non-switched memory B**	-	-	-	-
**CD19+CD27+IgD− class-switched memory B**	-	-	-	-
**CD19+CD27−IgD+ naïve**	-	-	-	-
**CD19+CD5+ Bregs**	-	-	-	-

At month 3 the profound reduction in total CD3−CD19+ B cells left too few B-cell events to reliably resolve switched memory, unswitched memory, naïve and Bregs subpopulations by flow cytometry.

S3b. T-cell subsets

T-cell subset (%)	All (N=35)	Non-resp. (N=12)	Resp. (N=23)	P

T-cell subset (%)	All (N=35)	Non-resp. (N=12)	Resp. (N=23)	P
**CD3+**	71.75±4.8	72.9±4.8	71.1±4.75	0.29
**CD3+CD4+**	39.4±7.12	39.36±7.2	39.52±6.11	0.69
**CD3+CD8+**	20.9±6.51	20.9±.5.62	20.9±6.46	0.92
**CD4/CD8 ratio**	2.34±0.51	2.35±0.1	2.34±0.1	0.76
**Tregs CD4+CD25+FoxP3+**	4.12±1.16	3.19±0.64	4.6±1.1	**<0.001**
**CD4+CD45RA+ naïve**	24±7.25	23.4±6.5	24.3±7.7	0.75
**CD4+CD45RO+ memory**	31.3±8	29.4±8.8	32.3±7.6	0.33
**CD45RA+**	40.5±8.7	37.2±5.8	42.2±9.6	0.1
**CD45RO+**	41.8±8.63	43.9±8.2	40.7±8.8	0.3
**CD45RA+RO+ transitional**	3.1±1.3	3.17±1.55	3.01±1.25	0.73

S3c. Monocyte/NK subsets and SIRI

Parameter	All (N=35)	Non-resp. (N=12)	Resp. (N=23)	P
**WBC (/µL)**	8377±1324	8680±1069	8219±1436	0.33
**Lymphocytes (%)**	21.7±1.2	21.6±1.15	21.8±1.2	0.57
**Lymphocytes (/µL)**	1256±126.8	1263±123.1	1252±128.4	0.29
**Monocytes (/µL)**	693.5±30.5	687.1±28	697.5±31.7	0.34
**Monocytes (%)**	8.23±0.15	8.14±0.12	8.27±0.14	0.06
**CD14++CD16− classical (%)**	80±2.6	79.3±2.88	80.2±2.44	0.32
**CD14++CD16+ intermediate (%)**	7.5±1.4	8.96±0.7	6.75±1	**<0.001**
**CD14+CD16++ non-classical (%)**	6.5±2	6±2.34	6.7±1.8	0.28
**CD14+HLA-DR+ activated (%)**	86.15±4.65	88.3±4.3	85±4.4	0.08
**SIRI**	1.36±0.16	1.48±0.16	1.3±0.13	**<0.001**
**NK cells CD16+CD56+ (%)**	11.1±3.29	10.9±3.27	11.1±4.28	0.114

Shaded rows denote P ≤ 0.05.

Table S4. Month-6 immunophenotypic subsets.

S4a. B-cell subsets

B-cell subset (%)	All (N=35)	Non-resp. (N=12)	Resp. (N=23)	P
**Total CD3−CD19+ (residual)**	1.81±0.40	2.95±0.5	1.22±0.19	**<0.001**

B-cell subset (%)	All (N=35)	Non-resp. (N=12)	Resp. (N=23)	P
**CD19+CD27+IgD+ non-switched memory B**	-	-	-	-
**CD19+CD27+IgD− class-switched memory B**	-	-	-	-
**CD19+CD27−IgD+ naïve**	-	-	-	-
**CD19+CD5+ Bregs**	-	-	-	-

At month 6 the profound reduction in total CD3−CD19+ B cells left too few B-cell events to reliably resolve switched memory, unswitched memory, naïve and Bregs subpopulations by flow cytometry.

S4b. T-cell subsets

T-cell subset (%)	All (N=35)	Non-resp. (N=12)	Resp. (N=23)	P
**CD3+**	71.3± 5.4	71± 5.7	71.4± 5.4	0.84
**CD3+CD4+**	44.7 (43.1–48.3)	44.1 (42.8–48.6)	44.9 (43.4–48.9)	0.222
**CD3+CD8+**	20.3 (19.7–22)	20.1 (19.4–22)	20.5 (19.1–22.5)	0.345
**CD4/CD8 ratio**	2.05 ± 0.34	2.32 ± 0.25	1.97 ± 0.32	**0.002**
**Tregs CD4+CD25+FoxP3+**	6.2± 1	5.38± 0.73	6.6± 0.83	**<0.001**
**CD4+CD45RA+ naïve**	22.8 ± 7.15	22.2 ± 7.95	23.2 ± 6.86	0.715
**CD4+CD45RO+ memory**	33.1 (26.5–37.2)	33.5 (26.1–37.9)	32.9 (25.1–38.2)	0.101
**CD45RA+**	36.9 ± 7.6	35.9 ± 7.95	37.4 ± 7.6	0.599
**CD45RO+**	38.9 ± 7.6	37.2 ± 6.5	39.8 ± 8.2	0.318
**CD45RA+RO+ transitional**	2.93 ± 1.1	3.04 ± 1.15	2.88 ± 1.1	0.699

S4c. Monocyte/NK subsets and SIRI

Parameter	All (N=35)	Non-resp. (N=12)	Resp. (N=23)	P
**WBC (/µL)**	8650 (7740–8880)	8630 (7850–8780)	8660 (7810–8750)	0.704
**Lymphocytes (%)**	20.7 ± 1.3	21.2 ± 1.54	20.5 ± 1.13	0.781
**Lymphocytes (/µL)**	1740 ± 149	1751 ± 174	1735 ± 138	0.159
**Monocytes (/µL)**	658.8 ± 29.5	654.4 ± 31	661.2 ± 29	0.535
**Monocytes (%)**	7.9 (7.7–8.2)	7.8 (7.5–8.3)	8 (7.4–8.4)	0.133
**CD14++CD16− classical (%)**	79.2 (77.1–81.9)	78.5 (75.2–81.1)	80.5 (76.1–82.3)	0.345
**CD14++CD16+ intermediate (%)**	5.25± 1.56	6.37 ± 0.7	4.67 ± 1.5	**<0.001**

Parameter	All (N=35)	Non-resp. (N=12)	Resp. (N=23)	P
**CD14+CD16++ non-classical (%)**	5.2 (3.1–8.4)	4.9 (3.4–8.6)	5.4 (2.9–8.5)	0.159
**CD14+HLA-DR+ activated (%)**	86.7± 4.23	84.7± 3.88	87.7± 4.12	0.05
**SIRI**	1.87± 0.41	1.9± 0.48	1.85± 0.38	0.78
**NK cells CD16+CD56+ (%)**	11.2 (10.1–11.5)	11.5 (10–12.2)	11.2 (10.7–11.8)	0.470

Shaded rows denote $P \leq 0.05$.

S5. Antibody panels and corresponding fluorochrome assignments

Tube	FITC or Alexa Fluor 488	PE	PerCP	APC
1	CD3–FITC	CD16 + CD56	CD45	CD19
2	CD3–FITC	CD4	CD45	CD8
3	CD45RA–FITC	CD45RO	CD45	CD4
4	HLA-DR–FITC	CD14	CD45	—
5	CD16–FITC	CD14	CD45	—
6	IgD–FITC	CD27	---	CD19
7	FoxP3–Alexa Fluor 488	---	CD4	CD25
8	CD5–FITC	CD19	CD45	—

Supplementary Figure Legends

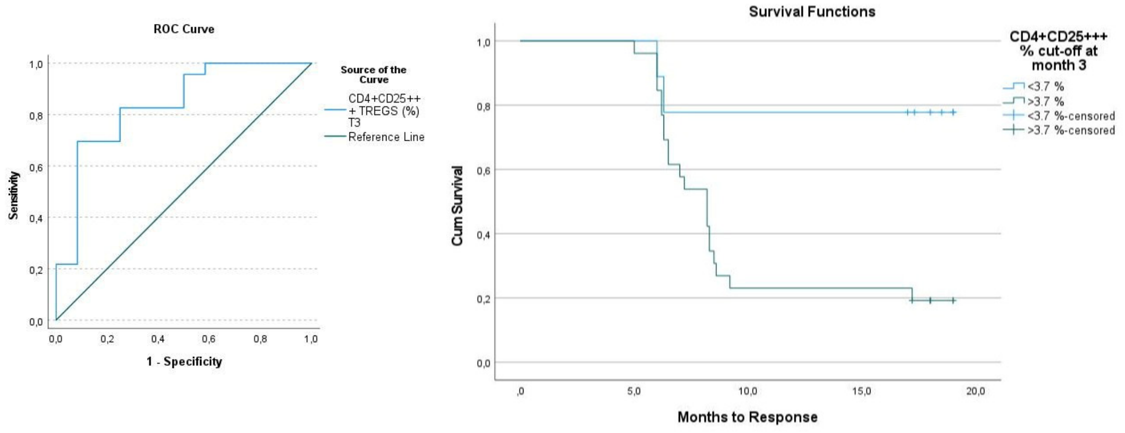

Figure S1. Tregs CD4+CD25+FoxP3+ (%) at month 3 — ROC and Kaplan–Meier analyses. AUC 0.84 (95% CI 0.69–0.98), P <0.001; sensitivity 82%, specificity 75%.

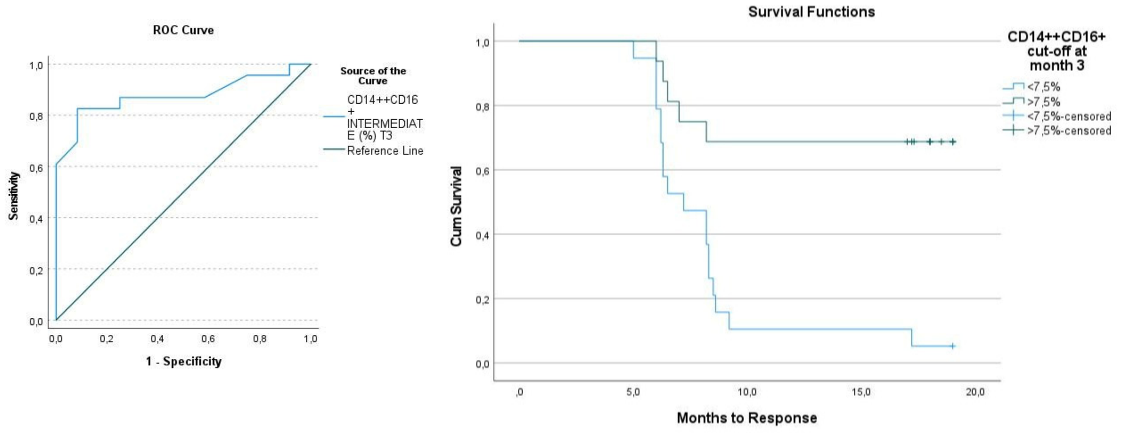

Figure S2. Intermediate CD14++CD16+ monocytes (%) at month 3 — ROC and Kaplan–Meier analyses. AUC 0.87 (95% CI 0.76–0.99), P <0.001; sensitivity 82%, specificity 84%.

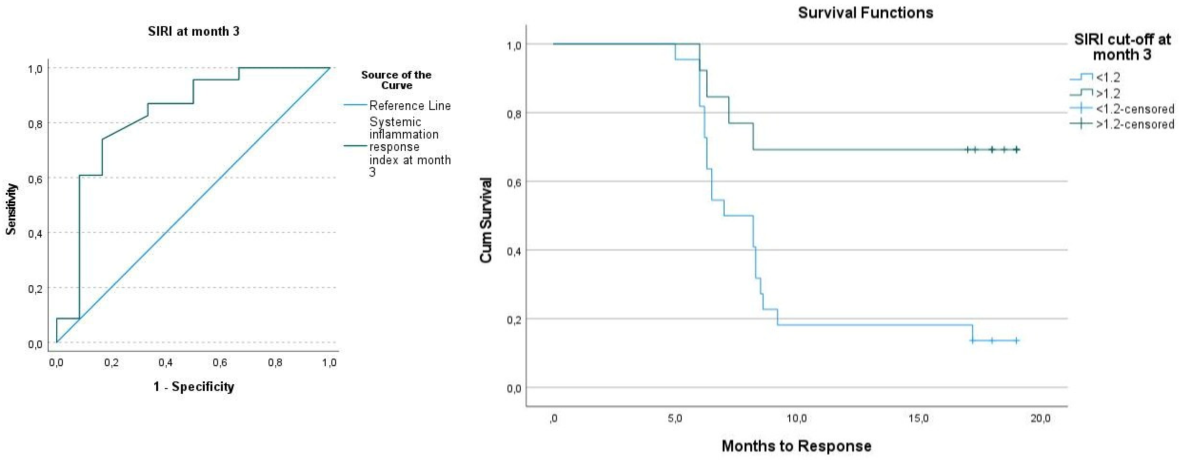

Figure S3. SIRI at month 3 — ROC and Kaplan–Meier analyses. AUC 0.82 (95% CI 0.66–0.96), P = 0.004; sensitivity 86%, specificity 82%.

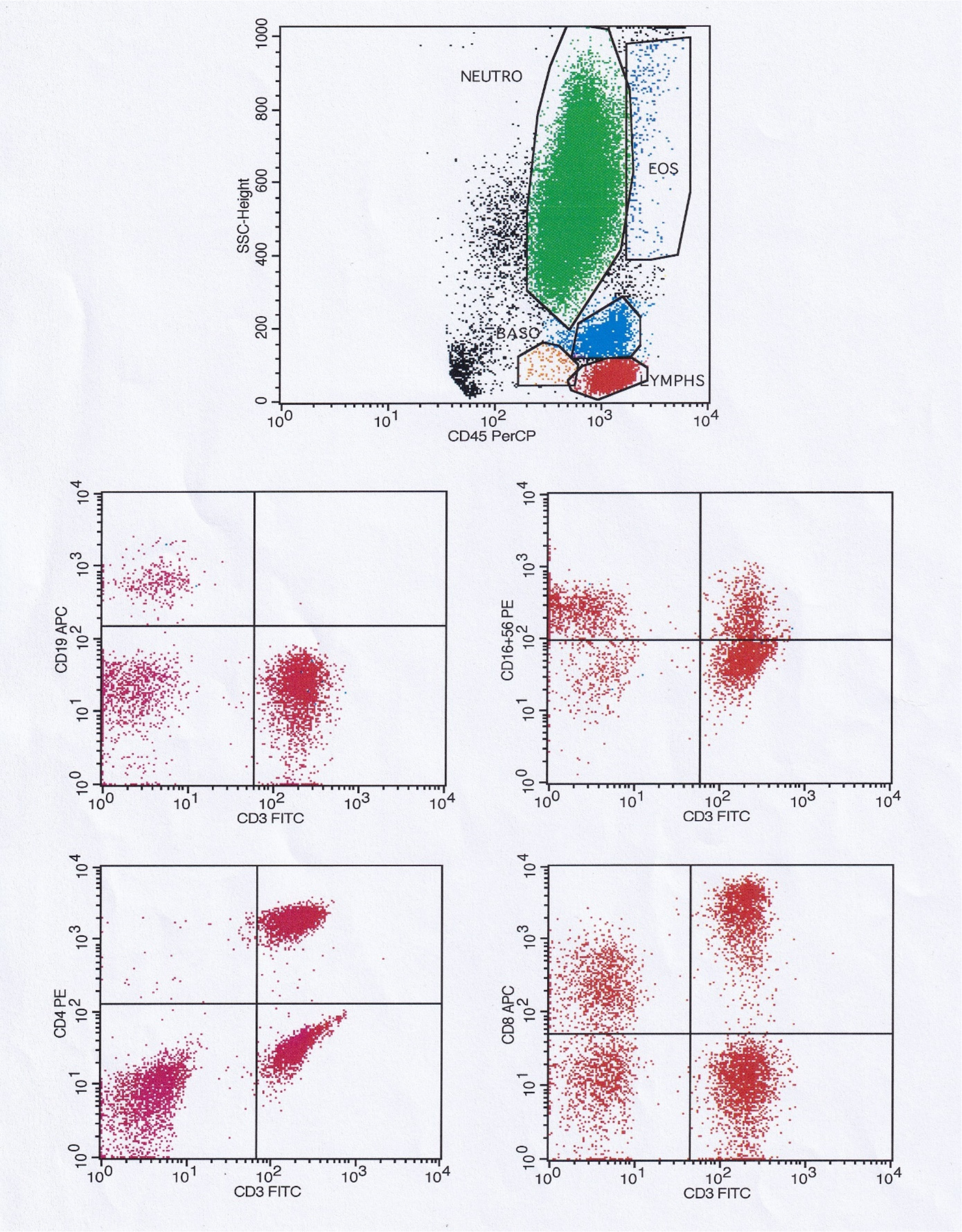

Figure S4. Representative dot plots depicting lymphocyte gating (red colour) with B-lymphocytes, T-lymphocytes, and natural killer (NK) cells defined as CD16+CD56+ cells, CD4+ T cells, and CD8+ T cells.

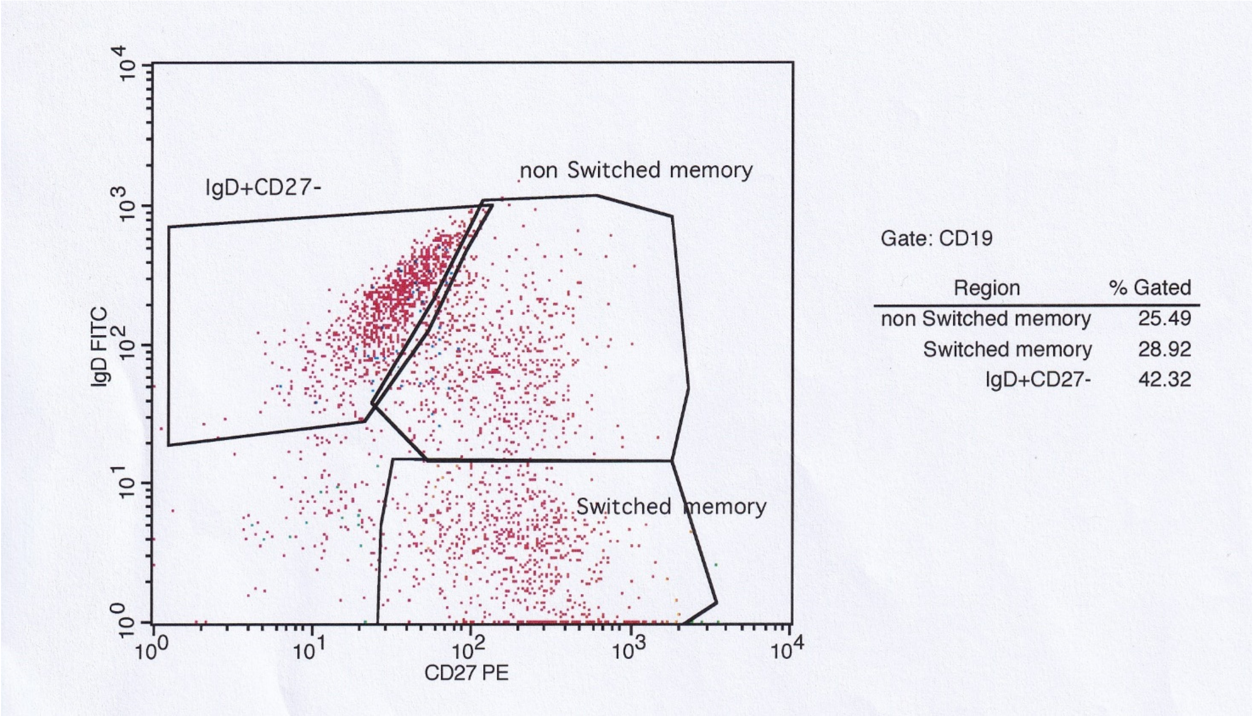

Figure S5. Representative dot plots depicting B-lymphocytes sub-populations, class-switched memory, unswitched memory and naïve B-cells.

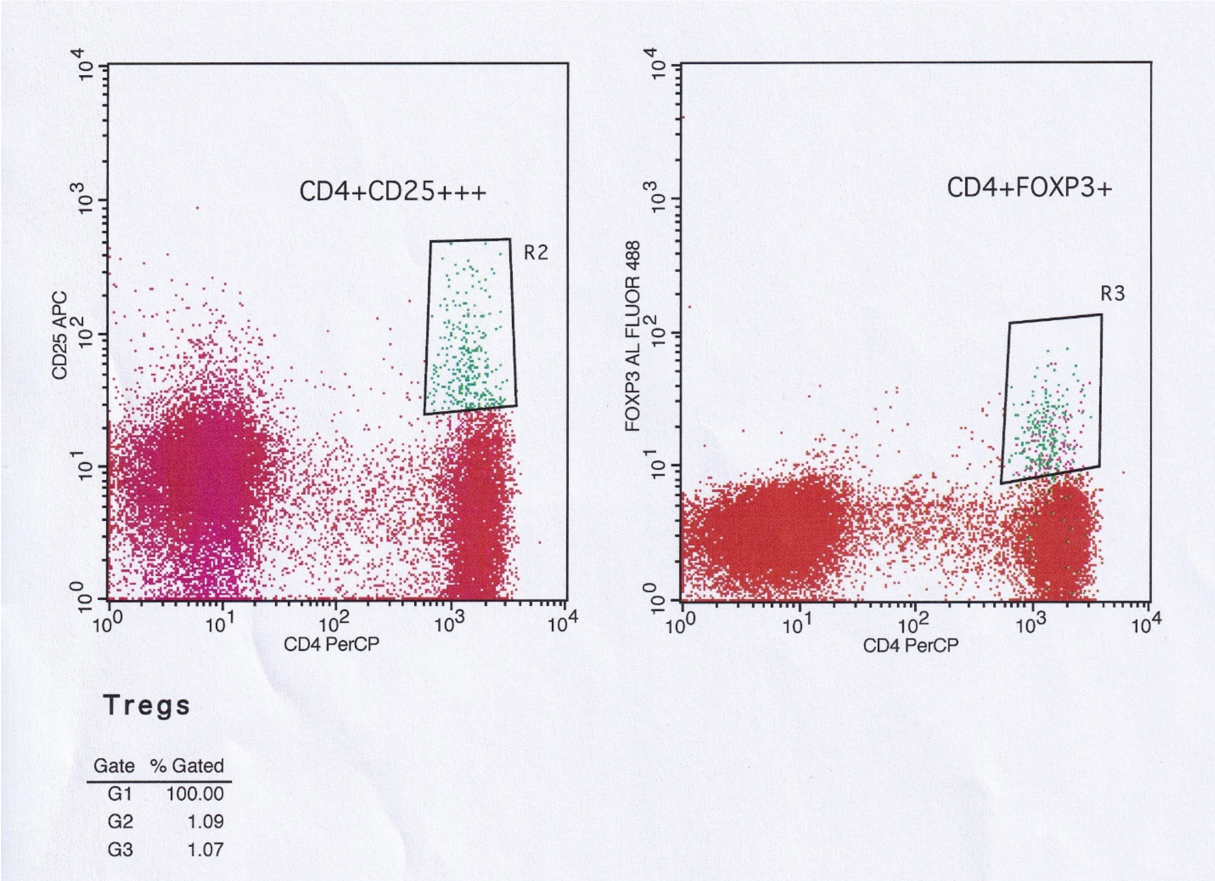

Figure S6. Representative dot plots depicting T regulatory cells (Tregs) defined as CD4+ FoxP3+ CD25^{high}.
